# Development of an Analytical Assay for Electrochemical Detection and Quantification of Protein-Bound 3-Nitrotyrosine in Biological Samples and Comparison with Classical, Antibody-Based Methods

**DOI:** 10.3390/antiox9050388

**Published:** 2020-05-06

**Authors:** Ksenija Vujacic-Mirski, Kai Bruns, Sanela Kalinovic, Matthias Oelze, Swenja Kröller-Schön, Sebastian Steven, Milos Mojovic, Bato Korac, Thomas Münzel, Andreas Daiber

**Affiliations:** 1 Center for Cardiology, Department of Cardiology 1–Molecular Cardiology, University Medical Center, 55131 Mainz, Germany; ksenija.vujacic.mirski@gmail.com (K.V.-M.); sanelakalinovic@gmail.com (S.K.); matthias.oelze@unimedizin-mainz.de (M.O.); swenja.kroeller-schoen@gmx.de (S.K.-S.); sesteven@uni-mainz.de (S.S.); tmuenzel@uni-mainz.de (T.M.); 2 Institute of Clinical Chemistry and Laboratory Medicine, Medical Center of the Johannes Gutenberg University, 55131 Mainz, Germany; kai.bruns@unimedizin-mainz.de; 3 Faculty of Physical Chemistry, University of Belgrade, Studentski trg 12-16, 11000 Belgrade, Serbia; milos@ffh.bg.ac.rs; 4 Institute for Biological Research “Sinisa Stankovic”—National Institute of Republic of Serbia, University of Belgrade, 11000 Belgrade, Serbia; b.korac@bio.bg.ac.rs; 5 Partner Site Rhine-Main, German Center for Cardiovascular Research (DZHK), Langenbeckstr. 1, 55131 Mainz, Germany

**Keywords:** oxidative stress, peroxynitrite, protein-bound 3-nitrotyrosine, HPLC with electrochemical detection, mitochondrial superoxide

## Abstract

Reactive oxygen and nitrogen species (RONS) cause oxidative damage, which is associated with endothelial dysfunction and cardiovascular disease, but may also contribute to redox signaling. Therefore, their precise detection is important for the evaluation of disease mechanisms. Here, we compared three different methods for the detection of 3-nitrotyrosine (3-NT), a marker of nitro-oxidative stress, in biological samples. Nitrated proteins were generated by incubation with peroxynitrite or 3-morpholino sydnonimine (Sin-1) and subjected to total hydrolysis using pronase, a mixture of different proteases. The 3-NT was then separated by high performance liquid chromatography (HPLC) and quantified by electrochemical detection (ECD, CoulArray) and compared to classical methods, namely enzyme-linked immunosorbent assay (ELISA) and dot blot analysis using specific 3-NT antibodies. Calibration curves for authentic 3-NT (detection limit 10 nM) and a concentration-response pattern for 3-NT obtained from digested nitrated bovine serum albumin (BSA) were highly linear over a wide 3-NT concentration range. Also, ex vivo nitration of protein from heart, isolated mitochondria, and serum/plasma could be quantified using the HPLC/ECD method and was confirmed by LC-MS/MS. Of note, nitro-oxidative damage of mitochondria results in increased superoxide (O_2_^•–^) formation rates (measured by dihydroethidium-based HPLC assay), pointing to a self-amplification mechanism of oxidative stress. Based on our ex vivo data, the CoulArray quantification method for 3-NT seems to have some advantages regarding sensitivity and selectivity. Establishing a reliable automated HPLC assay for the routine quantification of 3-NT in biological samples of cell culture, of animal and human origin seems to be more sophisticated than expected.

## 1. Introduction

Oxidative stress is reported to be a hallmark of almost all neurodegenerative and cardiovascular diseases [1,2,3]. Clinical trials support a role of oxidative stress for cardiovascular prognosis [4,5]. Cellular oxidative stress conditions are defined by the increased formation of reactive oxygen and nitrogen species and/or impaired cellular antioxidant defense system, depletion of low molecular weight antioxidants, and a shift in the cellular redox balance [6,7], which is associated with oxidative damage of biomolecules such as proteins [8,9]. A prominent example is the nitration, e.g., by peroxynitrite (PN) of Tyr34 in mitochondrial superoxide dismutase (MnSOD) [10,11,12], which is associated with its inhibition and the pathogenesis of various diseases [13,14,15]. 

There is increasing evidence that redox modifications of proteins can affect enzyme activities and thus represent alterations of the cellular signaling network (reviewed in [16,17,18,19]). Protein tyrosine nitration represents a prominent posttranslational redox modification and is associated with a broad range of different diseases [3,20,21]. Besides reports on unspecific protein tyrosine nitration, used as a general marker of nitro-oxidative stress, there are reports on site-specific nitrations with a direct impact on enzymatic activities and properties [22]. Examples are the very rapid tyrosine nitration and inactivation of prostacyclin synthase, a P450 protein, by nanomolar concentrations of PN [23,24], which was later postulated to involve heme-thiolate catalysis and a ferryl intermediate [25,26]. A similar metal-catalyzed mechanism was postulated for MnSOD that facilitates PN-mediated nitration and dimerization of tyrosine residues, leading to inactivation of the enzyme [10,11,12]. Of note, the apoenzyme and zinc-substituted enzyme showed significantly decreased rate constants for the reaction with PN and did not catalyze the nitration of phenolic compounds [10]. More examples are presented in Section 4 to support the biological importance of this oxidative post-translational modification.

Levels of 3-NT, mostly caused by PN in vivo formation, allow the indirect quantification of triumvirate ^•^NO, O_2_^•–^, and PN, which is of great importance for the regulation of vascular tone but also represents a great challenge, since these species are short-lived. Therefore, the aim of the present study was to establish an HPLC assay for the electrochemical detection of 3-NT in biological samples and to compare this method with different immunological methods (enzyme-linked immunosorbent assay (ELISA) and dot blot analysis).

## 2. Materials and Methods

### 2.1. Chemicals

Pronase from *Streptomyces griseus* (lyophylized powder) was obtained from Roche (Mannheim, Germany). Sin-1, hydrochloride was obtained from Cayman Chemical Company Michigan, USA; 3-NT standard was obtained from Sigma, Merck KGaA, Darmstadt, Germany. D3-3NT standard was obtained from Toronto Research Chemicals, Toronto, Canada. Anti-nitrotyrosine, rabbit immunoaffinity purified IgG was obtained from EMD Merck Millipore Corp, Merck KGaA, Darmstadt. Peroxidase labeld anti-rabbit IgG (H&L) affinity purified, made in goat was obtained from Vector Laboratories, CA, USA. Triphenylphosphonium-linked dihydroethidium (mitoSOX) was purchased from Invitrogen/Thermo Fischer Scientific, Waltham, MA, USA. PN was prepared by quickly adding one after another: 0.6 M potassium nitrate and 1.5 M potassium hydroxide in the previously mixed solution of 0.6 M hydrochloric acid and 0.7 M hydrogen peroxide. 

### 2.2. Animals Handling and Euthanasia

All animals were treated in accordance with the Guide for the Care and Use of Laboratory Animals as adopted by the U.S. National Institutes of Health and approval was granted by the Ethics Committee of the University Hospital Mainz and the Landesuntersuchungsamt Rheinland-Pfalz (Koblenz, Germany; permit number: 23 177-07/G 18-1-001). Male Wistar rats (6 weeks old, 300 g, Charles River Laboratories, Sulzfeld, Germany) and male C57BL/6 mice (13 ± 3 weeks) were used for the study and all efforts were made to minimize suffering. Only male animals were used because we usually always use this gender for our vascular function studies. Due to hormonal differences, vascular function would turn out differently between male and female animals (e.g., specifically depending on the menstrual cycle). Animals were killed under isoflurane anesthesia by transection of the diaphragm and exsanguination (for plasma generation). Heart and liver were harvested for further analysis. As a model of type 2 diabetes mellitus (T2DM), we used previously harvested kidney tissue from Zucker Diabetic Fatty (ZDF-Lepr^fa/fa^) rats that were previously obtained from Charles River at an age of 16 ± 1 weeks and fed with Purina 5008 chow as described [27].

### 2.3. Nitration of Bovine Serum Albumin (BSA) or Biological Samples

Purified BSA was used at a final concentration of 1 mg/mL in 0.1 M potassium phosphate buffer pH 7.4. Blood was obtained by heart puncture directly after the addition of heparin to the heart. A small amount of the blood was then mixed with 10% 50 mM tri-potassium ethylenediaminetetraacetic acid (EDTA) for the final concertation of 5 mM and centrifuged 10 min 1452× *g*. Plasma (supernatant) was taken, frozen in liquid nitrogen, and kept at −80 °C until use. Protein count of plasma was determined by Lowry method and it was diluted to 0.6 mg/mL protein in 0.1 M potassium phosphate buffer pH 7.4 before digestion. 

For the isolation of mitochondria, a published protocol was used [28,29]. Briefly, cardiac and liver tissues underwent homogenization in HEPES buffer (4-(2-hydroxyethyl)-1- piperazineethanesulfonic acid; composition in mM: 50 HEPES, 70 sucrose, 220 mannitol, 1 EGTA (ethylene glycol-bis(β-aminoethyl ether)-*N*,*N*,*N*′,*N*′-tetraacetic acid), and 0.033 bovine serum albumin) and centrifugation at 1500× *g* for 10 min at 4 °C, followed by another centrifugation step of the supernatant at 2000× *g* for 5 min (pellets were not used). Next, centrifugation of the supernatant at 20,000× *g* for 20 min was applied, the pellet was collected, and a suspension in 1 mL of HEPES buffer was prepared. The suspension was centrifuged again at 20,000× *g* for 20 min, but this time, a suspension of the pellet was prepared in 1 mL of Tris buffer (composition in mM: 10 Tris, 340 sucrose, 100 KCl, and 1 EDTA). The resulting mitochondria-enriched suspensions containing 5–10 mg/mL of total protein (according to Lowry assay) were kept at 0 °C, were all adjusted to a similar protein content (based on the lowest determined concentration).

A small aliquot of PN (80 mM in 0.1 M NaOH) was added by rapid mixing of the reaction solutions (protein homogenate in potassium phosphate 100 mM buffer) and was allowed to completely decompose within 5 min. Sin-1 (100 mM from a 0.1 M acidic stock solution) was added to the protein solutions and incubated for 90 min at 37 °C to allow complete decomposition. 

### 2.4. Dot Blot Analysis for Protein-Bound 3-Nitrotyrosine

Analysis of total protein homogenates and of plasma samples was performed by dot blot as previously described [30,31]. Briefly, 50 µL (1 µg/µL protein based on Bradford analysis) of the heart homogenate or EDTA plasma were transferred to a Protran BA85 (0.45 µm) nitrocellulose membrane (Schleicher&Schuell, Dassel, Germany) by a Minifold I vacuum Dot-Blot system (Schleicher&Schuell, Dassel, Germany). Each slot was washed twice with 200 µL phosphate buffered saline (PBS) before and after protein transfer. The membrane was dried for 60 min at 60 °C. Equal loading of protein amounts per dot was then verified by staining the membrane with Ponceau S. Next, the membrane was incubated with blocking buffer and then primary antibody in blocking buffer according to the supplier’s instructions. Protein tyrosine nitration was detected using a specific antibody for 3-NT (1:1,000, anti-nitrotyrosine, rabbit immunoaffinity purified IgG, EMD Merck Millipore Corp, Merck KGaA, Darmstadt Germany). Positive bands were detected by enhanced chemiluminescence after incubation with a peroxidase-coupled secondary antibody (1: 5000, peroxidase-conjugated goat anti rabbit antibody) (Vector Laboratories, CA, USA). All incubation and washing steps were performed according to the manufacturer’s instructions. Densitometric quantification of the dots was performed using the Super Signal enhanced chemiluminescence kit from Thermo Scientific using a ChemiLux Imager (CsX-1400M, Intas, Göttingen, Germany) and Gel-Pro Analyzer software (Media Cybernetics, Bethesda, MD, USA).

### 2.5. ELISA Quantification of Protein-Bound 3-Nitrotyrosine

Levels of 3-nitrotyrosine were determined in nitrated BSA samples using a commercial ELISA kit (OxiSelect™ Nitrotyrosine ELISA, Cell Biolabs, San Diego, CA, USA) following the instructions of the vendor.

### 2.6. HPLC/ECD and UV/Vis Detection of 3-Nitrotyrosine

Pronase digestion was performed as previously described for bacterial monooxygenase-3 (P450_BM-3_) and camphor 5-monooxygenase (P450_CAM_) [26,32]. Briefly, all the samples were diluted to the desired protein amount in 0.1 M potassium phosphate buffer pH 7.4 containing 1 mM CaCl_2_ for the stabilization of the proteases during digestion at 37 °C. For different types of samples (heart, plasma, mitochondria), adjustments were made regarding the percentage of acetonitrile (which is used for protein denaturation and for better solubility of the liberated amino acid), as well as final pronase concentration and total time of the digestion. Nitrated solution of 1 mg/mL BSA was prepared with 5 *v/v*% acetonitrile and incubated with total 2 mg/mL of pronase with multiple additions over 24 h at 37 °C. The digestion of lipophilic proteins may take 3 days, whereas for other hydrophilic proteins such as BSA, it was already completed within 2–3 h after the addition of pronase [33]. Heart proteins (final 0.6 mg/mL proteins) were prepared with 20 *v/v*% acetonitrile and plasma (0.6 mg/mL proteins) and mitochondria (0.1 mg/mL proteins) with 10 *v/v*% acetonitrile. Because of the interference in the HPLC and a smaller amount or protein solution used, plasma and mitochondria were incubated with the final concentrations of 1 mg/mL and 0.5 mg/mL pronase respectively, which was added by multiple additions over 48 h period of time, while BSA and hearts were digested by 2 mg/mL pronase over 24 h. All digested samples were freed from residual proteins by centrifugation through 10 kDa Microcon centrifugal filter device from Millipore Corporation (Bedford, USA) and the eluates were measured immediately. Several experiments have been conducted to ensure that, under these conditions, hydrolysis was completed within the indicated time scale.

Kidney probes of the control and ZDF rats were digested by a specific protocol according to a recently published protocol [34]. Kidney tissue was glass-glass homogenized in 100 mM potassium phosphate buffer and after Lowry protein determination samples were diluted to a final protein concentration of 10 mg/mL. Samples were placed on ice for 10 min in 10% (*v/v*) ice cold trichloroacetic acid (TCA, 1M). Then, 75% (*v/v*) of 0.1 M ice cold TCA was added and the mixture was incubated for another 20 min. Samples were vortexed after both additions of TCA. Samples were then centrifuged for 30 min at 20,000× *g*, the supernatant was removed, and the pellet was washed and resuspended with 500 µL of pure acetone, which was followed by 10 min centrifugation at 20,000× *g*. The supernatant was carefully removed, and the pellet was resuspended in 100 mM potassium phosphate for a further 36 h digestion with 3 mg/mL pronase as described above.

For some samples, aliquots (100 µL) were analyzed on a HPLC system with UV/Vis detection according to a slightly modified method as previously described [25]. Briefly, the HPLC system was purchased from Jasco (Groß-Umstadt, Germany) with a typical composition (control unit, two pumps for high pressure gradient, high pressure mixer, UV/VIS and fluorescence detectors, and an autosampler (AS-2057 plus with 4 °C cooling device). The generation of gas bubbles from the solvents that can cause an unstable detection baseline was prevented using a degasser unit. For the separation of the product and reactant mixtures, a reversed-phase column was used (C_18_-Nucleosil 100-3 (125 × 4), Macherey & Nagel, Düren, Germany). Optimal separation was achieved by the application of a high pressure gradient with acetonitrile as the organic/nonpolar component and citrate buffer as the aqueous/polar component (50 mM, pH 2.2) of the mobile phase. The following percentages of the organic solvent were applied: 0–7 min, 8 *v/v*%; 8–9 min, 90 *v/v*%; 10 min, 8 *v/v*%. The flow was 1 mL/min and 3-NT was detected by its absorption at 360 nm.

For the electrochemical detection of 3-NT UltiMate 3000 system with Dionex™ CoulArray™ (Coulometric Array Detector) (Thermo Fisher Scientific GmbH, Dreieich, Germany) was used, which is high-quality instrument designed for the detection of electroactive species. The system was controlled by two different software programs: Chromeleon Chromatography Management System (Chromeleon) software and CoulArray software. When starting the system, pumps were first purged, then pump pressure was equilibrated for at least 1 h. The samples were loaded to the autosampler and the sampling settings were also controlled by Chromeleon software (amount of sample taken, speed, washing of the needle). The CoulArray software controls two parts of the HPLC/ECD system: the chamber for the column where temperature was assigned before starting the sequence and the electrochemical detector. Detector consisted of two coulometric electrochemical channels which can use up to 4 different cells each. A coulometric cell with a large surface area consisting of porous graphite electrode allows complete oxidation (or reduction) of the electroactive species minimizing the noise and providing enhanced sensitivity. The Coularray method is shorter than the Chromeleon method and starts first by turning the cells on to the assigned voltage, then it performs autozero and waits for the injection signal by Chromeleon software. Each sample run ends with a short cleaning procedure of the electrochemical cells (setting all cells to 800mV). For detection of 3-NT potentials of 0, +150, +300, +450, +600, +650, +700, and +800 mV were used and 3-NT peek was observed between 650 and 800 mV with the most pronounced signal at 800 mV. HPLC separation was done using Phenomenex column (Kintex^®^ 2.6 µm C18 100Å LC Column 100 × 4.6 mm) (Aschaffenburg, Germany) and analysis was done at 27 °C with 20 µL of sample. Changes in temperature can shift the chromatographic peaks. Mobile phase consisted of 26.3 mM sodium citrate and 10.9 mM sodium acetate, although different pH and methanol percentages were used to achieve better separations and detection in the different types of samples. 

BSA samples were measured with isocratic elution of 1 mL/min with a mobile phase consisting of 2.8 *v/v*% methanol in citrate/acetate buffer and a pH of 4.75, and under these conditions the 3-NT peak showed a retention time (RT) of 6.2 min. Heart proteins were analyzed with isocratic elution of 1.3 mL/min with a mobile phase consisting of 3.25 *v/v*% methanol in citrate/acetate buffer and a pH of 4.85 (3-NT at RT = 4.39 min). Plasma proteins were measured with isocratic elution of 1.3 mL/min with the mobile phase consisting of 3 *v/v*% methanol in citrate/acetate buffer and a pH of 4.95 (3-NT at RT = 4.76 min). All mitochondrial samples were measured with isocratic elution of 1 mL/min with a mobile phase consisting of 3.5% methanol in citrate/acetate buffer and a pH of 3.75 (3-NT at RT = 4.05 min). Kidney tissue samples were measured with isocratic elution of 0.75 mL/min with a mobile phase consisting of pure citrate/acetate buffer and a pH of 3.75 (3-NT at RT = 7.03 min). 

### 2.7. Detection of Mitochondrial Superoxide Formation by mitoSOX HPLC Method and Plate Reader Assay 

Mitochondrial oxidative stress by superoxide was also measured by a modified HPLC-based method to quantify triphenylphosphonium-linked 2-hydroxyethidium (2-OH-mito-E+) levels as previously described [35,36]. Mitochondrial suspensions were further diluted to the final protein concentration of 0.1 mg/mL in 0.5 mL of PBS buffer containing mitoSOX (5 µM) and then incubated for 15 min at 37 °C. After the incubation step, 50 *v/v*% of acetonitrile was added in order to destroy the mitochondrial membrane and extract the mitoSOX oxidation products, samples were subjected to centrifugation and the resulting supernatant was subjected to HPLC analysis (100 µL per sample injection). The HPLC system and reversed-phase column were the same as those used for 3-NT quantification. Optimal separation was achieved by application of a high pressure gradient with acetonitrile as the organic/nonpolar component and citrate buffer as the aqueous/polar component (50 mM, pH 2.2) of the mobile phase. The following percentages of the organic solvent were applied: 0 min, 22 *v/v*%; 10 min, 50 *v/v*%; 22 min, 63 *v/v*%; 23–25 min, 100 *v/v*%; 25–27 min, 22 *v/v*%. The flow was 0.5 mL/min and mitoSOX was detected by its absorption at 360 nm whereas triphenylphosphonium-linked ethidium (mitoE+) and 2-OH-mito-E+ were detected by fluorescence (Ex. 500 nm/Em. 580 nm). 

The mitochondrial supernatant was also used for the plate reader assay. Here, 200 µL of supernatant was pipetted in the 96 well black plate (Berthold Technologies), and the fluorescence was measured by Mithras^2^ chemiluminescence/fluorescence plate reader with double monochromator (Berthold Technologies) using the same fluorescence parameters as described for the HPLC method above.

### 2.8. LC-MS/MS Analysis

LC-MS/MS analysis was carried out in positive ion mode on a Waters Xevo TQ-XS triple quadrupole mass spectrometer coupled to a Waters Acquity UPLC (Waters, Eschborn, Germany) consisting of a binary UPLC pump equipped with a degasser, an autosampler and a column oven. MassLynx 4.2 software was used for instrument control, data acquisition and data processing. Chromatographic separation was performed using a Waters Acquity UPLC BEH C18 column (1.7 μm 2.1 × 50 mm). Injection volume was 5 µL. A gradient was applied using a mobile phase 0.1 *v/v*% formic acid in water (A) and 0.1 *v/v*% formic acid in acetonitrile (B) at 40 °C and a flow rate of 0.6 mL/min with the following percentages of (B): 0 min, 3 *v/v*%; 2 min, 20 *v/v*%; 2.01 min, 95 *v/v*%; 2.2 min, 95; 2.21 min, 3 *v/v*%. Total run time was 2.5 min. The first 0.6 min. of the gradient was directed to waste to reduce contamination of the mass spectrometer. Optimized ion source parameters were as follows: Capillary voltage 0.8 kV, cone voltage 20 V, desolvation temperature 600 °C, desolvation gas flow 1200 L/h, source temperature 150 °C, source gas flow 150 L/h. Argon was used as collision gas. Mass transitions were monitored at *m/z* 227 → 117 (Collison energy: 18 eV) and *m/z* 227 → 181 (10 eV) for 3-NT and *m/z* 230 → 119 (18 eV) and *m/z* 230 → 184 (10 eV) for D3-3-NT. MS/MS-spectra of 3-NT were recorded scanning *m/z* from 80 to 230.

### 2.9. Statistical Analysis

Results are expressed as mean ± SD. One-way ANOVA (with Bonferroni’s correction for comparison of multiple means) or, where appropriate, the equivalent non-parametric Kruskal-Wallis test (Dunn multiple comparison) was used for comparisons of ROS detection and oxidative protein modification (SigmaStat for Windows, version 3.5, Systat Software Inc.). *p* values < 0.05 were considered statistically significant.

## 3. Results

### 3.1. Comparison of the Detection and Quantification of 3-NT Standards as Well as Nitrated BSA Standards by HPLC/ECD, Dot Blot and ELISA 

**Figure 1:**
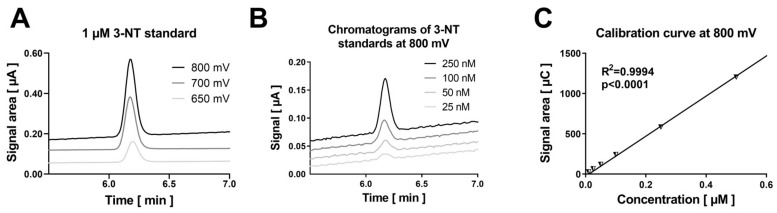
Detection of authentic 3-NT standards by HPLC/ECD. (**A**) The coulometric signal for 3-NT increased for the applied potentials from 650 to 800 mV as shown by the representative chromatograms. (**B**) The sensitivity of the HPLC/ECD analysis was good and a concentration of 25 nM 3-NT was easily detectable as shown by the representative chromatograms. (**C**) The calibration curve was highly linear over a concentration range of 10–500 nM. Analysis was carried on with 20 µL of sample at 27 °C with isocratic elution using a flow of 1 mL/min and a mobile phase consisting of 26.3 mM sodium citrate and 10.9 mM sodium at pH 4.75 with 2.8 *v/v*% methanol (RT of 3-NT was observed at 6.2 min).

**Figure 2:**
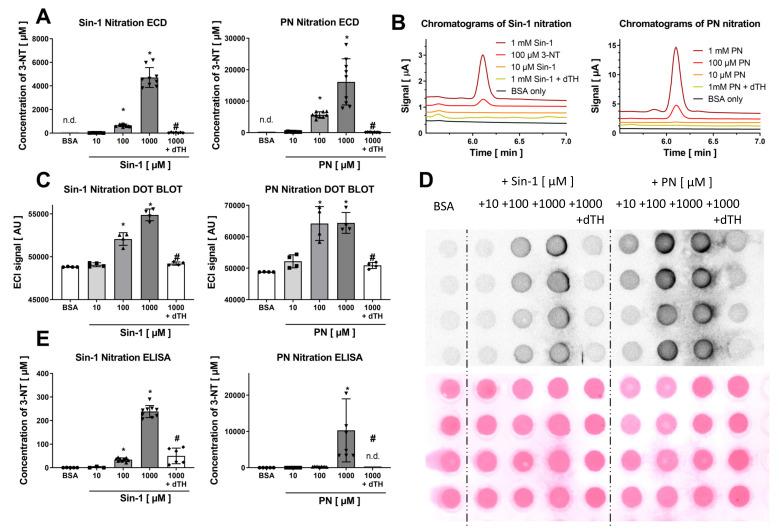
Detection of free 3-NT from nitrated BSA by HPLC/ECD and comparison with detection of BSA-bound 3-NT by antibody-based methods. Purified BSA was nitrated by Sin-1 or PN (10–1000 µM) and generated 3-NT was reduced by dTH. These samples were subjected to pronase digest before HPLC/ECD analysis (**A**) or were not digested for quantification by dot blot (**C**) and ELISA (**E**). (**B**) Representative chromatograms are shown for the HPLC/ECD quantification. (**D**) Representative blots are shown for the dot blot quantification. Equal protein loading was checked by Ponceau staining of the membrane. HPLC/ECD analysis was performed with 20 µL of sample at 27 °C with isocratic elution (1 mL/min, mobile phase: 26.3 mM sodium citrate and 10.9 mM sodium at pH 4.75 with 2.8 *v/v*% methanol; 3-NT eluted at 6.2 min). Data are presented as mean ± SD of n = 9 (A), 9 (**C**) and 4 (**E**) independent experiments. * indicates *p* < 0.05 versus BSA untreated control group; ^#^ indicates *p* < 0.05 versus 1000 µM Sin-1/PN group.

Coulometric detection of 3-NT yielded the most pronounced signal when an electrode potential of 800 mV was applied and allowed proper detection of 25 nM authentic 3-NT (Figure 1A,B). The calibration curve showed good linearity over a 3-NT standard concentration range between 10 and 500 nM (Figure 1C), which was still highly linear up to a concentration of 10 µM 3-NT. Purified BSA was nitrated using Sin-1 and PN and was afterwards digested using pronase. Digested samples were used for the detection by HPLC/ECD while parts of undigested samples were used for the detection by dot blot analysis or ELISA. Quantification of the free 3-NT signal from nitrated BSA showed a concentration-dependent increase with Sin-1 or PN, which was absent if nitrated samples were treated with the potent reductant dithionite (dTH) (Figure 2A,B). Dithionite is known to reduce 3-NT to 3-aminotyrosine. In untreated BSA, no 3-NT signal could be detected. Dot blot analysis using a specific 3-NT antibody showed a comparable pattern but a quite substantial background with untreated BSA samples or those nitrated and then treated with dTH (Figure 2C,D). In addition, there was plateau formation of the 3-NT signal in the presence of the middle and highest PN concentrations. The ELISA showed a nice concentration-nitration signal correlation but an unexpected signal pattern as well as large signal variation for the PN-treated BSA samples (Figure 2E). Overall, the HPLC/ECD method provided the best sensitivity for both nitrating agents, and for Sin-1, the best correlation between Sin-1 concentration and 3-NT yield as well as the most reliable effect of dTH reduction of 3-NT.

### 3.2. Detection and Quantification of Free 3-NT from Nitrated Tissue Homogenates and Plasma Samples by HPLC/ECD as Well as Comparison with HPLC/UV or ELISA 

**Figure 3:**
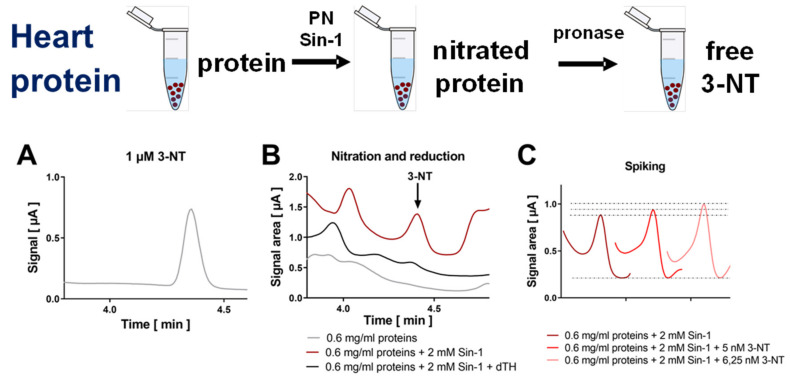
Detection of free 3-NT from nitrated heart proteins by HPLC/ECD. Heart homogenates were nitrated by Sin-1 and generated 3-NT was reduced by dTH. These samples were subjected to pronase digest before HPLC/ECD analysis. (**A**) Representative chromatogram of authentic 3-NT standard. (**B**) Representative chromatograms of Sin-1 treated heart proteins after digest with or without dTH. (**C**) Spiking of potential 3-NT peak with low concentrations of authentic 3-NT standard to proof the identity of this peak. HPLC/ECD analysis was performed with 20 µL of sample at 27 °C with isocratic elution (1.3 mL/min, mobile phase: 26.3 mM sodium citrate and 10.9 mM sodium at pH 4.85 with 3.25 *v/v*% methanol; 3-NT eluted at 4.39 min).

**Figure 4:**
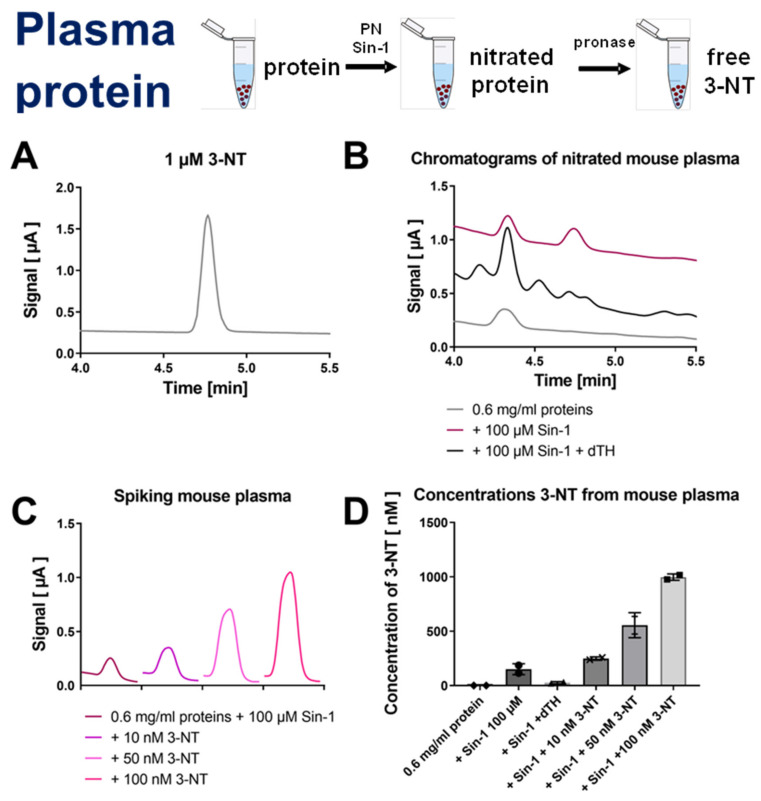
Detection of free 3-NT from nitrated plasma proteins by HPLC/ECD. Plasma was nitrated by Sin-1 and generated 3-NT was reduced by dTH. These samples were subjected to pronase digest before HPLC/ECD analysis. (**A**) Representative chromatogram of authentic 3-NT standard. (**B**) Representative chromatograms of Sin-1 treated plasma proteins after digest with or without dTH. (**C**) Spiking of potential 3-NT peak with low concentrations of authentic 3-NT standard to proof the identity of this peak. (**D**) Quantification of 3-NT yield of experiments shown in panels (**B**,**C**). HPLC/ECD analysis was performed with 20 µL of sample at 27 °C with isocratic elution (1.3 mL/min, mobile phase: 26.3 mM sodium citrate and 10.9 mM sodium at pH 4.75 with 3 *v/v*% methanol; 3-NT eluted at 4.76 min).

**Figure 5:**
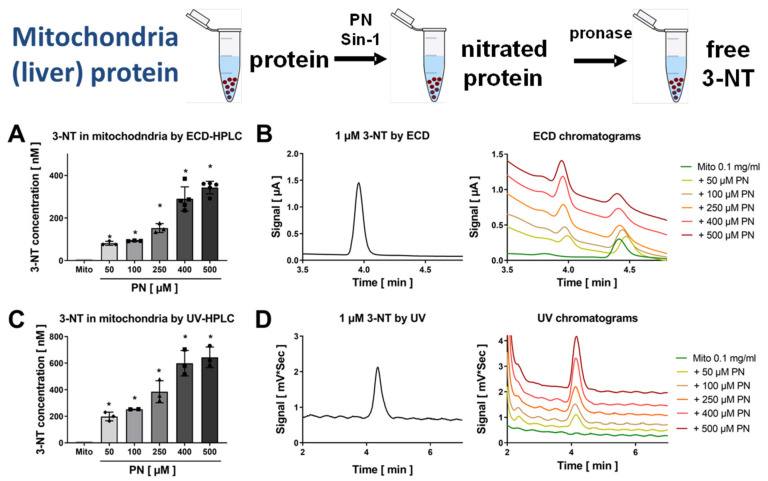
Detection of free 3-NT from nitrated liver mitochondria by HPLC/ECD or HPLC/UV. Isolated liver mitochondria were nitrated by PN (50–500 µM). These samples were subjected to pronase digest before HPLC/ECD analysis (**A**) or HPLC/UV analysis (**C**). Representative chromatograms are shown for the HPLC/ECD quantification (**B**) or the HPLC/UV quantification (**D**). HPLC/ECD analysis was performed with 20 µL of sample at 27 °C with isocratic elution (1 mL/min, mobile phase: 26.3 mM sodium citrate and 10.9 mM sodium at pH 3.75 with 3.5 *v/v*% methanol; 3-NT eluted at 4.05 min). Data are presented as mean ± SD of n = 3–5 (**A**) and 3–5 (**C**) independent experiments. * indicates *p* < 0.05 versus Mito untreated control group.

Hearts were homogenized, nitrated by Sin-1, and then the homogenate was digested using pronase and subjected to HPLC/ECD analysis (Figure 3). The resulting coulometric signal showed a similar retention time as compared to 3-NT standard, was lost upon treatment with dTH and showed symmetric peak increase after spiking with low concentrations of authentic 3-NT standard (Figure 3A–C). Likewise, plasma samples were nitrated by Sin-1, the samples were then digested using pronase and subjected to HPLC/ECD analysis (Figure 4). The resulting coulometric signal showed a similar retention time as compared to 3-NT standard, was lost upon treatment with dTH and showed symmetric peak increase after spiking with low concentrations of authentic 3-NT standard (Figure 4A–D). Liver mitochondria were isolated, nitrated by PN, and the mitochondria were sonicated and digested using pronase and subjected to HPLC/ECD or HPLC/UV analysis, whereas for dot blot analysis and ELISA, the digestion step was omitted (Figure 5). Mitochondrial protein showed a good concentration-nitration signal correlation when HPLC/ECD analysis was applied (Figure 5A,B). However, HPLC/UV analysis revealed no disadvantage as compared to the coulometric detection (Figure 5C,D). The reason for the different retention times of 3-NT in the chromatograms of the different biological samples is that the mobile phase conditions had to be slightly modified for each cell organelle, tissue, or plasma in order to achieve the proper separation of the 3-NT peak from other contaminating compounds (see figure legends and Section 2 for a description of the different mobile phase conditions).

### 3.3. Detection and Quantification of Free 3-NT from Tissue Samples of Diabetic Rats with or without Combined Sin-1 Nitration by HPLC/ECD as Well as Comparison with LC-MS

**Figure 6:**
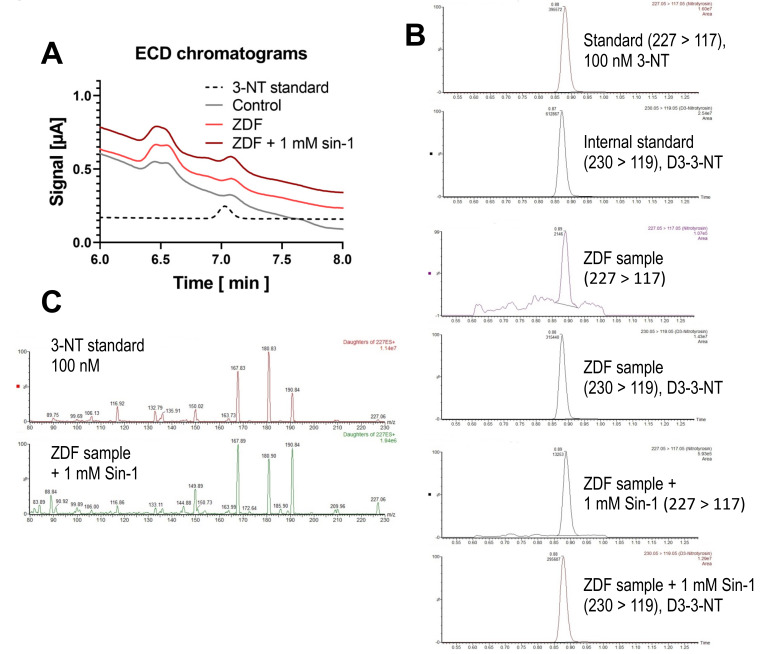
Detection of free 3-NT from control and diabetic animals with and without combined nitration by Sin-1. Kidney homogenates of healthy control and diabetic (ZDF) rats (10 mg/mL protein) were subjected to pronase digest before HPLC/ECD analysis or LC-MS/MS analysis. A special digestion protocol was used (see Methods for kidney samples). HPLC/ECD analysis was performed with 40 µL of sample at 27 °C with isocratic elution (0.75 mL/min, mobile phase: 26.3 mM sodium citrate and 10.9 mM sodium at pH 3.75; 3-NT eluted at 7.03 min). Representative chromatograms are shown for the HPLC/ECD quantification (**A**) or LC-MS/MS analysis (**B**). LC-MS/MS analysis was performed after adding an identical amount of deuterated 3-NT (D3-3-NT) to compensate for matrix effects. Mass transitions monitored at 227 → 117 (3-NT) and 230 → 119 (D3-NT) are shown. Representative MS/MS spectra of precursor ion at *m/z* = 227 are shown for the 3-NT standard (100 nM) and the sample from ZDF rat with 1 mM Sin-1 treatment (**C**).

A small 3-NT signal was observed using HPLC/ECD detection in the sample of the healthy control rat (33.8 nM 3-NT). The 3-NT signal was marginally higher in the sample of the diabetic (ZDF) rat (42.9 nM 3-NT) and was substantially increased upon treatment with Sin-1 (84.8 nM 3-NT) (Figure 6A). The signal in the diabetic rat sample was confirmed using LC-MS/MS (Figure 6B) and MS/MS-spectra (Figure 6C). For LC-MS/MS analysis an identical amount of a deuterated internal standard of 3-nitrotyrosine (D3-3-NT) was added to 100 nM authentic 3-NT standard or digested tissue samples to compensate for matrix effects occurring during the ionization process. An approximately 6-fold increase after treatment with Sin-1 was observed. The LC-MS/MS method used also offers the possibility of absolute quantification. Furthermore, the presence of 3-NT was confirmed by comparison of MS/MS fragmentation patterns of authentic 3-NT standard and in vivo samples of precursor ion at *m/z* = 227. Standard and in vivo samples showed only common MS/MS signals. Despite a reliable 3-NT signal in the in vivo samples, no differences between healthy control and diabetic rat samples could be observed by LC-MS/MS.

### 3.4. Correlation of Mitochondrial Nitration and Superoxide Formation in Response to PN Treatment 

**Figure 7:**
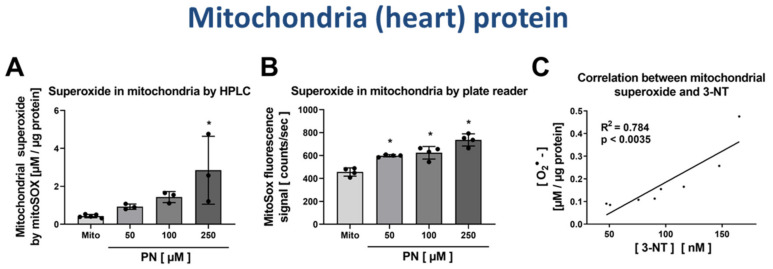
Detection of superoxide generation by mitochondria by mitoSOX HPLC and correlation with 3-NT levels. Isolated heart mitochondria were nitrated by PN at concentrations of 50–250 µM. These samples were split, one aliquot was used for measurement of mitochondrial superoxide formation using mitoSOX/HPLC and the other aliquot for determination of free 3-NT levels by HPLC/ECD after digestion. Mitochondrial superoxide formation was determined using HPLC-based quantification of 2-OH-mito-E+ (**A**) and ROS formation was measured using a fluorescence plate reader assay for the mitoSOX oxidation products (**B**). The yield of mitochondrial 3-NT was correlated with superoxide formation rate for the different PN-treated samples (**C**). HPLC/ECD analysis was performed with 20 µL of sample at 27 °C with isocratic elution (1 mL/min, mobile phase: 26.3 mM sodium citrate and 10.9 mM sodium at pH 3.75 with 3.5 *v/v*% methanol; 3-NT eluted at 4.05 min). Data are presented as mean ± SD of n = 3–5 (**A**); n = 4 (**B**) and n = 8 (**C**) independent experiments. * indicates *p* < 0.05 versus Mito untreated control group.

Mitochondria were isolated from rat hearts and nitrated by PN at three increasing concentrations. Mitochondrial superoxide formation was measured by mitoSOX HPLC method or plate reader assay and showed a good correlation between PN concentration used for the nitration/oxidation of mitochondria and superoxide formation rate (Figure 7A,B). As the 3-NT yield from digested nitrated mitochondria was also determined for the same PN-treated samples, we were able to correlate the mitochondrial superoxide formation rate with the 3-NT concentrations. There was a good linear correlation between mitochondrial superoxide formation rate and the 3-NT concentrations in the same sample (Figure 7C), suggesting that oxidative damage of mitochondria (3-NT but probably also thiol oxidation) initiates mitochondrial superoxide formation, e.g., by pro-oxidative state of respiratory complexes. This would be in line with the previously suggested crosstalk concept among different ROS sources [37,38,39,40]. 

## 4. Discussion

With the present study, we show that HPLC/ECD quantification is suitable for detection of free 3-NT from purified BSA and protein homogenates subjected to total hydrolysis by pronase after in vitro nitration by Sin-1 and PN. Concentration–response-curves of 3-NT standards were highly linear (detection limit 10 nM with 20 µL injection volume corresponding to 200 fmol). The presence of 3-NT in nitrated bovine serum albumin standards was validated by other methods (ELISA and dot blot analysis based on specific 3-NT antibodies). With our data, we also show that protein-bound 3-NT not only represents a footprint of PN formation and marker of oxidative stress but also correlates with increased mitochondrial superoxide formation rates, pointing towards self-propagating oxidative stress vicious circles and ROS-induced ROS formation as previously reported [37,38,41,42,43]. We also found 3-NT in samples of diseased (diabetic) animals using HPLC/ECD as well as LC-MS/MS measurement, however, with only marginal increase over 3-NT content in healthy control animals. Therefore, translation of our in vitro biological assay to the in vivo situation seems more sophisticated than expected.

### 4.1. Importance of the Quantification of Oxidative Stress in General and 3-Nitrotyrosine in Particular 

The importance of reliable quantification of 3-NT in biological samples, as a read-out of nitric oxide and superoxide balance, is given by the close connection between oxidative stress and cardiovascular prognosis, which is supported by a number of small cohort clinical studies. For example, the differential effects of vitamin C infusion on flow-mediated dilation (FMD) in coronary artery disease patients with high or low burden of ROS formation are associated with cardiovascular prognosis [44] and the impairment of FMD in 52 smokers versus controls is associated with lower blood levels of reduced glutathione [45]. This connection is also supported by a correlation between improvement of FMD and higher superoxide dismutase activity as well as a correlation between impairment of FMD in 59 patients with chronic kidney disease versus controls and higher oxidized low-density lipoprotein (oxLDL) or asymmetric dimethyl-L-arginine (ADMA) levels [46]. Finally, patients with sleep apnea versus controls (n = 69) show negative correlations between vascular function (reactive hyperemia index) and oxidative stress markers in blood (malondialdehyde and 8-oxo-deoxyguanosine) [47].

Under physiological conditions, cells produce low levels of O_2_^•–^ which can be largely increased by numerous stimuli such as inflammatory processes [48], hypoxia-reoxygenation (ischemia-reperfusion) [49] and aging [50,51] involving several O_2_^•–^ sources. Major sources of O_2_^•–^ are phagocytic and non-phagocytic NADPH oxidases, xanthine oxidase, mitochondria, and an uncoupled nitric oxide synthase, that might interact and stimulate each other in a crosstalk fashion [37,38]. Besides the direct toxic effects of O_2_^•–^ and higher hydrogen peroxide concentrations by disrupting Fe-S-clusters and oxidizing critical thiols in proteins as well as Fenton-type reactions, O_2_^•–^ reacts in a diffusion-controlled fashion with nitric oxide (^•^NO) resulting in PN formation [52]. Since formation of PN is at expense of ^•^NO, this reaction decreases the bioavailability of this potent vasodilator and antiaggregatory compound while shifting the balance between protective and proinflammatory/proatherosclerotic actions to the pathophysiology [53,54]. Previous data suggest that ^•^NO and O_2_^•–^ mainly influence the redox balance in living cells and account for oxidative, nitrative, and nitrosative stress [19,55,56,57], and represent a central redox regulatory system in the endothelium and vasculature controlling vascular tone [58].

### 4.2. Biological Consequences of Protein Tyrosine Nitration

There are various reports on protein tyrosine nitration regarding mechanisms of 3-NT formation, its specific detection as well as the pathophysiological consequences, especially in diseases related to oxidative stress [3,15,53,54,55,56,57,58]. For example, the nitration and modulation of activity of ERK1/2 and protein kinase B (Akt) play an important role in angiotensin-II triggered vascular complications [59,60]. Tyrosine nitration by PN was reported to proceed via a free radical based mechanism with intermediary formation of tyrosyl radicals [61], which is largely enhanced in the presence of carbon dioxide [59,60,61]. The tyrosine content of most proteins is around 3.2%, but not all of them are available for nitration. Secondary structure and the local environment are important factors determining the nitration site. Nitrated tyrosines are found in loop structures (near proline or glycine) as well as in proximity of a negative charge (e.g., glutamate or aspartate) [62,63]. The presence of free metal ions such as Cu^2+^, Fe^3+^, and Fe^2+^ as well as bound in complexes, especially metal porphyrins (like hemin), act as nitration catalysts, while the presence of sulfur-containing residues (cysteine or methionine) decreases the probability of nitration due to competitive reactions with PN [13]. In heme-containing proteins, the nitration of tyrosine is facilitated by the formation of ferryl intermediates [62,64], which was observed in prostacyclin synthase (PGIS) and prevented by an enzyme inhibitor that binds to the metal complex pocket [16]. 

Concerning the biological significance, PGIS activity was attenuated after nitration of Tyr430 by PN [64] and contributed to endothelial dysfunction [65]. Although the inhibition of MnSOD was not only associated with Tyr34 nitration, but could also involve dityrosine formation [11,12], it has been demonstrated that reaction with PN is catalyzed by the manganese cation [66]. Nitration of cytochrome c by PN lead to conformational changes going hand in hand with altered redox properties including increased peroxidatic activity, resistance to reduction by ascorbate and different behavior in rat heart mitochondria [66]. Also, some constituents of the mitochondrial respiratory chain are subject to PN-mediated nitration, such as interactions with mitochondrial ATPase (complex V) and components of the mitochondrial membrane (e.g., permeability transition pore) [15]. Nitration of fibrinogen and plasminogen was proposed to contribute to pro-thrombotic conditions [67,68,69,70]. However, protein Tyr nitration might also leave the enzyme function entirely unaffected. Therefore, various protein nitrations observed in models of endotoxemia or inflammation could just present markers of PN formation or activation of the peroxidase/nitrite/H_2_O_2_ pathway [71], without direct connection to the underlying pathophysiology [72,73]. However, even if nitration does not cause direct enzyme inhibition or altered function, posttranslational modification in form of nitro-groups may cause immune responses as immunoglobulins (autoantibodies) against nitrated proteins were identified in patients that predict the outcome and risk in [74,75].

### 4.3. Comparison of Previous Reports on Detection and Quantification of Protein Tyrosine Nitration

Most reports on 3-NT detection and quantification by HPLC with various electrochemical methods (coulometric and amperometric, different electrodes) are based on in vitro nitrated samples. Only few reports describe the detection of 3-NT in samples of animals with different in vivo treatments, however, often without showing the proper negative controls. Ishida et al. used HPLC-ECD detection of 3-NT and reported a detection limit of less than 10 fmol 3-NT standard as well as correlation of lipopolysaccharide (LPS) dosage and plasma 3-NT levels in mice [76]. Shigenaga et al. used GC/MS detection of derivatized ^15^N-labeled 3-NT (as acetyl-3-aminotyrosine) in protein lysates from activated RAW 264.7 macrophages upon incubation with ^15^N-L-arginine [77]. Acetyl-3-aminotyrosine was also detected by HPLC-ECD and compared with the GC/MS data. Ohshima et al. used HPLC-ECD after online reduction for the detection of 3-NT in BSA and human plasma samples, which were nitrated with different nitrating agents (0.1–10 mM), and reported a detection limit of 0.1 pmol [78]. Hensley et al. managed to detect 3-NT in glial cell cultures which were treated with interleukin (IL-1β) by using an HPLC-ECD method [79]. Sodum et al. described a highly sensitive HPLC-ECD method for the detection of 3-NT and reported a detection limit for 3-NT standards of 50 fmol, and of 0.1 pmol in biological samples of tetranitromethane-treated rats [80]. Nuriel et al. reported an HPLC-ECD assay for 3-NT detection in most healthy tissues, which was 100-fold more sensitive than UV/Vis detection of 3-NT [81] and was modified from previous protocols [82,83]. To eliminate interfering signals and additionally affirm complete oxidation of 3-NT at 800 mV, Crabtree et al. used an HPLC-ECD method (700, 800, 900 mV) for 3-NT detection in kidney proteins [84]. Kumarathasan et al. developed HPLC methods with amperometric–CoulArray to simultaneous analyze norepinephrine, epinephrine, L-3,4-dihydroxyphenylalanine (DOPA), dopamine, 3-nitrotyrosine, m-, o-, and p-tyrosines [85]. The detection limit was in the low pmol range with amperometry, and in the low fmol range for the CoulArray method. Commonly used antibodies for semi-quantitative detection of 3-NT in tissues offer a great sensitivity but exert also an epitope preference and cross-reactivity, which leads to under/overestimation and misinterpretation of biological protein nitration. ELISA assays for 3-NT detection are widely used in preclinical and clinical studies (for review, see [86]). However, the broad product specifications and multiple manufacturers hamper easy comparison of these results and certainly some of these ELISA assays may not be reliable for the use in biological samples. In contrast, total hydrolysis and subsequent HPLC-analysis offers an alternative method for quantification of 3-NT [61,62]. Pitfalls in hydrolysis include losses of 3-NT by partially reducing conditions and false positive results due to the presence of nitrite and its nitrosating properties during hydrolysis in concentrated acids or under acidic conditions as generated during the freezing process in certain buffers [87,88]. Therefore, pronase digestion has been recommended [18,62]. Some previous reports even validated the ELISA-based detection methods for 3-NT, e.g., by an HPLC assay [89] or by LC/MS technique [90]. Likewise, immunohistochemical 3-NT detection was validated by 2D-PAGE and LC-MS for plasma fibronectin [91] and atherosclerotic lesions [92].

### 4.4. Comparison of Theory and Our Empiric Data

A theoretical assumption is that most tyrosine residues which are nitrated are surface-exposed in proteins [62]. We found that extended time period of digestion increases the yield of 3-nitrotyrosine suggesting that some nitrated tyrosine residues may be buried and not easily accessible to pronase-dependent total hydrolysis. This may be in accordance with previous observations that non-surface-exposed tyrosines can also be efficiently nitrated under physiological conditions, when neighbored metal centers (e.g., as found in manganese-porphyrin, P450 or heme enzymes) catalyze the hemolysis of PN and subsequent nitration of the phenolic ring [25]. Another theory is that 3-NT can be reduced to 3-aminotyrosine during storage or total hydrolysis by trace metal contaminations or reducing proteins/biomolecules in the samples [93], which may be most pronounced for surface-exposed, accessible 3-NT residues. This reduction is mimicked by dTH that is used as a proof of 3-NT identity (if dTH leads to diminished signal it should be 3-NT). We potentially confirmed both of these theoretical aspects by using sodium dTH in highly nitrated samples and subsequent loss of 3-NT signals as well as by the lack of 3-NT signals in samples of animal disease models with confirmed high levels of 3-NT, e.g., in septic or diabetic animals due to inflammation-dependent nitric oxide and superoxide formation. The very weak 3-NT signals in these samples during HPLC/ECD analysis points towards unspecific degradation processes of 3-NT (as also speculated in Section 5), especially of surface-exposed residues, whereas the buried 3-NT groups may still yield enough signal with immunostaining-based methods. A final theoretical consideration that we have proven here is that very high concentrations of PN induce a vicious cycle of ROS-induced ROS formation, as previously shown for self-amplification of mitochondrial ROS formation [43,94], and eventually cell death. Our data indicate that the incubation of mitochondria with high, supra-physiological concentrations of PN leads to higher 3-NT levels, but also enhanced mitochondrial superoxide formation (with a nice linear correlation), suggesting a vicious cycle of “ROS-induced ROS”, e.g., by well-known nitration of Mn-SOD, inactivation of aconitase, oxidation of thiols, and other oxidative stress processes at the level of mitochondrial respiratory complexes, as we have postulated previously [37,38].

### 4.5. Strengths and Limitations of the Present Study

ECD detection of 3-NT standard has great advantage because it maintains linearity across a wide range of concentrations. Within first 4–7 min, 3-NT elutes, which allows for the analysis of many samples in a short period of time. The specificity of our assay is based not only on the retention time, but also on the oxidation potential of 3-NT, which is quite specific for each analyte that can be oxidized. This decreases the chance of generating false positive or false negative results. The sample preparation and digestion are done at neutral pH, so it is not aggressive for the sample. Therefore, artificial nitration as observed during acidic hydrolysis is not possible. The detection limit is quite low (200 fmol in our assay, others even reported 10 fmol). On the other hand, preparation of the samples needs at least 24 h incubation, which could result in the catalytic conversion of 3-NT to tyrosine or to 3-aminotyrosine as reported by us and colleagues recently [93]. Also, completeness of digestion could be a problem due to “buried” 3-NT in lipophilic cores and also due to autodigestion of pronase during the long incubation times. A solution for the latter problem (incomplete hydrolysis) would be longer digestion times and adding fresh pronase multiple times during the process. However, again at risk of “artificial” loss of 3-NT. A major limitation of the HPLC-ECD method is the appearance of interfering peaks (e.g., from other amino acids, low molecular weight antioxidants/messengers) that vary in different biological samples. Therefore, with high background noise, it is impossible to detect 3-NT in samples, which are not artificially nitrated. 

## 5. Conclusions

It seems to be a great challenge to reproducibly detect 3-NT in samples with biological nitration obtained from animals and humans. However, 3-NT from artificially nitrated samples can be reproducibly detected, also allowing a comparison with immunological detection methods (ELISA and dot blot). Strengths of our HPLC/ECD method were the high linearity over a wide concentration range, the low detection limit, the confirmation of 3-NT identity by dTH-dependent reduction and co-elution of the 3-NT sample peaks after spiking with authentic standards. A major draw-back of the present HPLC/ECD protocol seems to be the separation of the 3-NT peak in complex biological samples or potentially the degradation of 3-NT during the pronase-based digestion method. As a major result of the present study, by combining optical and ECD-based HPLC methods, we were able to quantify 3-NT and superoxide in the same mitochondrial sample and could establish a direct linear correlation between these two oxidative stress read-outs. Our aim to quantify 3-NT in the artificially nitrated samples was achieved, but still, our method seems unsuitable for the routine use for 3-NT detection in tissues and plasma samples of diseased animals. Future studies will be dedicated to the quantification of 3-NT in tissue samples of nitrate-tolerant, diabetic, hypertensive, and septic mice and rats, also including the comparison of different detection methods.

## Abbreviations

3-NT, 3-nitrotyrosine; Sin-1, 3-morpholino sydnonimine; PN, peroxynitrite; ROS, reactive oxygen species; Reactive oxygen and nitrogen species (RONS); MnSOD, manganese superoxide dismutase (mitochondrial isoform); HPLC/ECD, high performance liquid chromatography with electrochemical detection; enzyme-linked immunosorbent assay (ELISA) O_2_^•–^, superoxide; BSA, bovine serum albumin; mitoSOX, triphenylphosphonium-linked dihydroethidium; NOS, nitric oxide synthase; dTH, dithionite; CoulArray, coulometric array system; retention time (RT).
